# Vaporization Phase Transition in Cryogenic Liquid Oxygen Sealing Film on Spiral Groove Faces

**DOI:** 10.3390/ma17061443

**Published:** 2024-03-21

**Authors:** Junjie Chen, Chunhong Ma, Shaoxian Bai, Jing Yang

**Affiliations:** 1 Institute of Process Equipment and Control Engineering, College of Mechanical Engineering, Zhejiang University of Technology, Hangzhou 310014, China; zjut_chenjunjie@163.com (J.C.); yangjing@zjut.edu.cn (J.Y.); 2 School of Mechanical and Energy Engineering, Zhejiang University of Science and Technology, Hangzhou 310023, China; machunhong@126.com

**Keywords:** vaporization phase transition, liquid oxygen, spiral grooves, cryogenic condition

## Abstract

The property of vaporization phase transition in liquid oxygen face seals is a key factor affecting the stability of mechanical face seals in many fields, especially under cryogenic conditions. Here, a numerical model based on the saturated vapor pressure is established to investigate the vaporization phase transition property of liquid oxygen sealing film. The novelty of this model is to take the influence of heat transfer and face distortions into consideration at the same time. The pressure and temperature distributions as well as face distortions are calculated, and then the property of vaporization phase transition and sealing performance are analyzed. It is found that spiral grooves may lead to the complex film temperature distributions and irregular vaporization distributions. With the increase in seal temperature and decrease in seal pressure, the vaporization area extends from the low-pressure side to the grooves area, and the vaporization rate increases rapidly. The more important thing is that the vaporization often brings a drastic fluctuation and non-monotonic change in opening force. Specifically, with the increase inin seal temperature from 55 K to 140 K, the opening force fluctuates violently, and the fluctuation range is more than 50%, showing an obvious instability. Finally, this study provides a design range of pressure and temperature values for liquid oxygen face seals. In these ranges, this kind of face seals can have a stable operation, which is beneficial to the practice engineering related to the complex properties of sealing fluid.

## 1. Introduction

Vaporization is a key factor affecting the performance and reliable operation of mechanical face seal, causing the seal to “suddenly burst” and excessive leakage, or leading to catastrophic collapse [1,2,3,4], which is essentially the phase change in liquid with pressure and temperature. Along with application of cryogenic liquid oxygen and hydrogen in liquid rocket engine, the problem of vaporization becomes increasingly prominent due to shear heat under high working speed of the seal [5,6,7,8]. Especially, for cryogenic liquid spiral groove face seals, the vaporization not only led to overheating [9], but also surface wear [10].

Lots of relevant works on vaporization have been published in past decades of years. After an experimental report on phase transition of liquid-lubricated contact mechanical face seals [1], published theoretical works have been carried out to analyze and modeling vaporization. Intermittent boiling model by Hughes [11], two thermal boundary models of isothermal and adiabatic heat by Hughes and Chao [12], continuous boiling model by Yasuna and Hughes [13], and two-phase mixed lubrication by Etsion [14,15] and Ruan [16] have been developed to analyze the vaporization of liquid film in smooth face seals. In these published works, for smooth face seals, it was found that the profile of vaporization distribution at sealing interface was relatively regular and boiling radius has been often proposed as a parameter to characterize vaporization distribution in the face film.

With the development and application of non-contact face seals [17,18,19], geometric groove structure, such as spiral groove, T-groove, multi-pore, etc., has been widely applied in the design of seal face so as to obtain longer serving life. Meanwhile, the face grooves make the vaporization phase transition more complex since the heat transfer and pressure distribution of fluid lubrication film are closely related to the geometric structure, which leads to the tendency of vaporization in the groove area [20,21]. In 1984, Shapiro et al. [9] theoretically analyzed a 50 mm diameter, spiral-groove face seal to seal liquid oxygen at 5.17 MPa pressure, and operating at surface speeds of 183 m/s. A pressure-balanced spiral-groove was proposed that circulates spiral-groove flow independent of leakage flow, which can preclude overheating from vaporization. However, the fluid temperature rise still affects the safe operation of the seal. Due to the extremely small viscosity at cryogenic temperature of the sealed fluid, the seals have to operate under poor lubricating conditions. In 2017, Zhang et al. [10] published an experimental work on cryogenic liquid nitrogen mechanical seal with spiral-groove face. The results show that in the speed-up stage, with rapidly increasing speed, the local face temperature rises dramatically to even higher than the vaporization temperature of liquid nitrogen. The temperature change may exceed 80 °C, even higher than 130 °C, when the speed quickly increases to 29,000 r/min. A two-phase flow phenomenon occurs, and serious point corrosion appeared on the stator surface.

In the researches on gas spiral groove face seal [22,23], it is found that, under the action of velocity shear, the maximum pressure distribution of lubricating fluid often appears at the end of the spiral groove, but the highest temperature appears at the outer diameter of the seal face. But, for liquid face seal, because of the high viscosity of the liquid, the temperature change in the fluid film in the groove area is more drastic than that of the gas seal. Meanwhile, low pressure induced phase transition often occurs in the spiral groove area, especially for the cases of high speed and low pressure [24,25]. Theoretically, it seems that the surface grooves will lead to significant effect on vaporization distribution.

The aim of this paper is to obtain the characteristics of vaporization phase transition of cryogenic liquid oxygen at spiral groove seal faces. A numerical model based on saturated vapor pressure is built considering heat transfer as well as face distortions. Then, the pressure and temperature distributions are calculated, and the influences of seal temperature on the vaporization distribution are discussed. Finally, vaporization distribution and sealing performance under different rotational speed, seal pressure and seal clearance are studied.

## 2. Model Development

**Figure 1:**
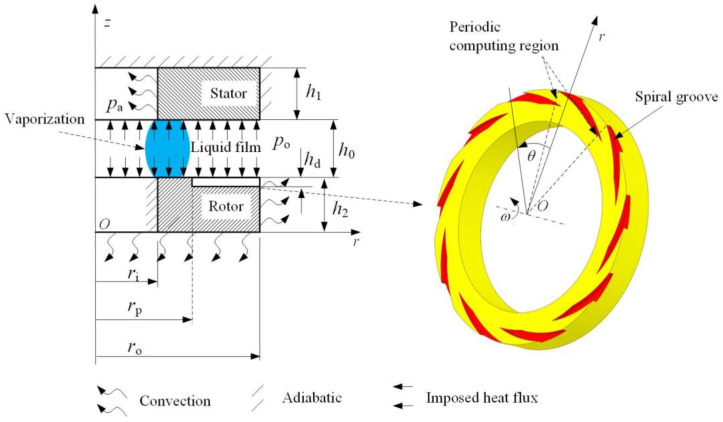
Diagram of liquid spiral groove face seal.

Figure 1 displays a typical structural diagram of liquid spiral groove face seal, where spiral grooves are designed on the rotor. At the rotational speed *ω*, the rotor and the stator are separated by a clearance *h*_0_. Under the shear of rotation speed, the grooves give rise to a hydrodynamic effect, which increases the opening force to keep the non-contact and stable running of the seal. At the low-pressure side, vaporization of liquid film often happens due to increase in temperature and decrease in pressure, which results in unstable running of the seal.

In the following numerical analysis of vaporization phase transition, the detailed structural parameters of the face seal are shown in Table 1.

### 2.1. Fluid Properties

Theoretically, vaporization is a type of phase change in fluid from a liquid state to gas state, which not only affected by pressure but also by temperature. In another words, the vaporization often happens when the pressure decreases to the saturated vapor pressure which depends on the temperature in some extent.

**Figure 2:**
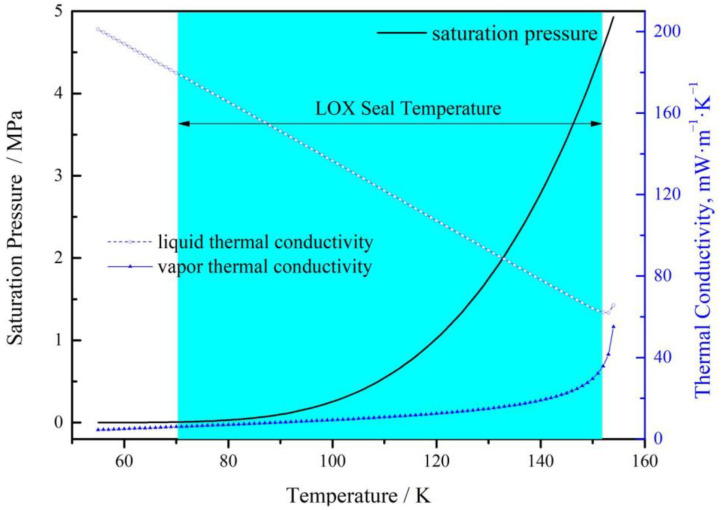
Change in saturation properties of liquid oxygen with increasing temperature.

**Figure 3:**
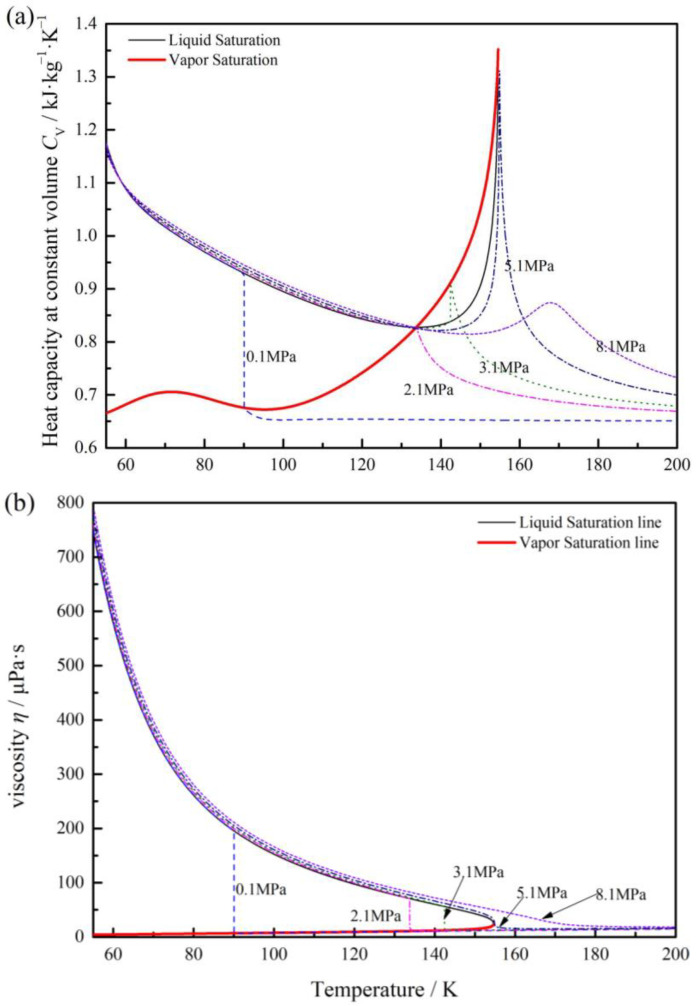
Variation thermo-physical properties of oxygen with temperature under different pressure: (**a**) specific heat capacity; (**b**) viscosity.

The thermo-physical properties of oxygen are obtained from NIST [26], and shown in Figure 2 and Figure 3. Figure 2 gives the saturation parameters such as saturation pressure, latent heat of vaporization, thermal conductivity of liquid oxygen and vapor oxygen.

As can be seen in this figure, when the temperature is lower than 110 K, the saturation pressure of oxygen increases from 178 Pa to 0.54 MPa, it means that the increase in temperature has little influence on the saturated vapor pressure under this condition. And then with the temperature continues to increase to 150 K, the saturated vapor pressure of oxygen increases rapidly to 4.2 MPa. This means that when the overall temperature is low, even if the pressure is reduced, the liquid oxygen is not easy to vaporization. However, at higher temperatures, vaporization of liquid oxygen will occur even at high fluid pressure.

Figure 2 also shows the thermal conductivity of liquid oxygen and vapor oxygen, the higher the temperature, the smaller the difference is. As the temperature increases from 70 K to 150 K, the thermal conductivity ratio of liquid/vapor oxygen decreases from 30 (179.7/6.0) to 2.16 (64.2/29.7). Hence, the thermal conduction effect cannot be ignored especially in low temperature in two phase face seal. Generally, in cryogenic engineering applications, liquid oxygen in the range of 70 K to 150 K is often used [5,6,7]. So, the vaporization phase transition in this temperature range is subsequently discussed.

The variation of oxygen specific heat capacity and viscosity with temperature under different pressure are shown in Figure 3. From Figure 3a, we can see the heat capacity, *C*_v_, decreases when phase transition from liquid oxygen to vapor under 2.1 MPa. Then, the pressure increases to critical point 5.4 MPa, the value shows fluctuation at the saturation point, as liquid oxygen, it decreases and then increases with the increase in temperature, the maximum value occurred in the saturation point; as vapor, it decreases rapidly. As the pressure continues to increase, this trend flattens out.

Figure 3b gives the variation of oxygen viscosity, it can be seen that once the liquid oxygen transition to vapor-phase, the viscosity decreases rapidly, especially in low pressure conditions under 5 MPa. As under the pressure of 2.1 MPa and temperature 109.2 K, the viscosity of liquid oxygen is 70.8 μPa·s, while the vapor is 10.9 μPa·s.

It means that once the phase transition, fluid properties show more complex changes, especially in low pressure under critical pressure. Then, the ideal gas law is not accurate in predicting the fluid and thermodynamic behaviors [27]. For the actual gas, specific heat capacity and viscosity must be considered [28]. It can also be seen that, the specific heat capacity and viscosity of gas oxygen present significant non-linear changes with increasing temperature and pressure, which means the loading capacity of the film may fluctuate irregularly with temperature and pressure, resulting in the instability of the sealing film.

### 2.2. Control Equations

Fluid lubrication based on the Reynolds equation has been wildly applied in analyzing of sealing performance. For vaporization phase transition analysis of face seals, the mathematical model mainly includes the Reynolds equation, energy equation, solid heat conduction equation and state equation.

The steady-state liquid Reynolds equation is expressed as

(1) $$\frac{\partial }{r\partial \theta }\left(\frac{\rho h^{3}}{\eta }\frac{\partial p}{r\partial \theta }\right)+\frac{\partial }{r\partial r}\left(\frac{r\rho h^{3}}{\eta }\frac{\partial p}{\partial r}\right)=6\omega \frac{\partial \left(\rho h\right)}{\partial \theta }$$

where *h* is the thickness of the lubricating film, *p* is the pressure of the lubricating film, *ω* is the rotating speed of the moving ring, *ρ* is the density of the lubricating medium, *r* and *θ* are the radials and circumferential coordinates of the selected calculation area, *η* is the viscosity of the lubricating medium.

Here, it is also assumed that once the vaporization pressure is reached, the liquid turns into vapor as discussed as the intermittent boiling model [11], so the vaporization regime is in a state of gas lubrication. Further, assuming the gas molecule as rigid spheres, according to the principle of energy equation, the energy per degree of motion freedom is equal to *E*_m_. Further, we assume that gas temperature only represents macroscopically inner energy of the gas molecular, so the following equation can be obtained [28]:

(2) $$T=\frac{i_{d}E_{m}}{c_{v}}$$

where, *i*_d_ is the freedom number of gas motions, and $c_{v}$ is specific heat at constant volume. Here, *i*_d_ = 5 for the oxygen gas.

Further, it is assumed that gas pressure is determined only by both translation energy of gas molecules and gas density. Hence, the pressure component induced by the gas can be expressed by

(3) $$p=\frac{R_{u}\rho i_{d}E_{m}}{c_{v}}$$

where *R*_u_ is the universal ideal gas constant, the value is 8.31434 J/(mol·K).

The energy equation of the sealing film can be modified to the following form [28]:

(4) $$\begin{array}{l} \left(\frac{h^{3}}{12\eta }\frac{\partial p}{r\partial \theta }-\frac{\omega rh}{2}\right)\frac{\partial T}{r\partial \theta }+\left(\frac{h^{3}}{12\eta }\frac{\partial p}{\partial r}\right)\frac{\partial T}{\partial r}=\\ -\frac{\eta \omega^{2}r^{2}}{h\rho c_{v}}+\frac{h^{3}}{12\eta \rho c_{v}}\left[{\left(\frac{\partial p}{r\partial \theta }\right)}^{2}+{\left(\frac{\partial p}{\partial r}\right)}^{2}\right]-\frac{k_{g,s1}}{\rho c_{v}}\left(T_{s1}-T\right)-\frac{k_{g,s2}}{\rho c_{v}}\left(T_{s2}-T\right) \end{array}$$

where, *k*_g,s1_ and k_g,s2_ are the thermal convective heat transfer coefficients at the interface of rotor and stator, respectively, *T*_s1_ and *T*_s2_ are the solid surface temperatures at the interface of rotor and stator, respectively.

The temperature *T*_s_ of seal rings is usually calculated by the heat conduction equation. The heat conduction equation of the rotor ring, Laplace equation, is

(5) $$\frac{\partial^{2}T_{s}}{r^{2}\partial \theta^{2}}+\frac{\partial }{r\partial r}\left(r\frac{\partial T_{s}}{\partial r}\right)+\frac{\partial^{2}T_{s}}{\partial z^{2}}=0$$

The heat conduction equation for stator ring, Laplace equation, is

(6) $$\frac{k_{c2}}{\rho_{s2}c_{s2}}\left[\frac{\partial^{2}T_{s}}{r^{2}\partial \theta^{2}}+\frac{\partial }{r\partial r}\left(r\frac{\partial T_{s}}{\partial r}\right)+\frac{\partial^{2}T_{s}}{\partial z^{2}}\right]=\omega \frac{\partial T_{s}}{\partial \theta }$$

where, *k*_c2_ is the thermal conductivity of the stator ring material. *ρ*_s2_, *c*_s2_ are the corresponding density and specific heat capacity.

### 2.3. Boundary Conditions

The following pressure boundary conditions are applied:

(7) $$p\left(r=r_{i},\theta \right)=p_{i}\;p\left(r=r_{o},\theta \right)=p_{o}\;p\left(r,\theta =\pi /N\right)=p\left(r,\theta =-\pi /N\right)$$

The axial dynamic temperature boundary conditions are

(8) $$T\left(r=r_{o},\theta \right)=T_{o}\;\mathrm{if}\;q_{r}\left(r=r_{o},\theta \right)<0\;T\left(r=r_{i},\theta \right)=T_{i}\;\mathrm{if}\;q_{r}\left(r=r_{i},\theta \right)>0\;T\left(r,\theta =\pi /N\right)=T\left(r,\theta =-\pi /N\right)$$

where $q_{r}=-\frac{h^{3}}{12}\frac{\partial p}{\partial r}$.

The parameters to measure the sealing performance mainly include the opening force *F*_o_ and the leakage rate *Q*. The dimensionless calculation formulas are

(9) $$F_{o}=\frac{1}{p_{a}r_{i}^{2}}\int_{0}^{2\pi }\int_{r_{i}}^{r_{o}}prdrd\theta$$

(10) $$Q=\frac{1}{h_{0}^{3}p_{a}}\int_{0}^{2\pi }h^{3}r\frac{\partial p}{\partial r}d\theta$$

To further characterize the vaporization phase transition in the sealed region, the phase transition ratio, *a*, is defined as follows.

(11) $$\alpha =\frac{\int_{0}^{2\pi }\int_{r_{i}}^{r_{o}}crdrd\theta }{\pi \left(r_{o}^{2}-r_{i}^{2}\right)}$$

where *c =* 1 if *p* < *p*_vapor_; otherwise *c =* 0.

To study the sealing performance, the dimensionless pressure is defined as $P=p/p_{a}$. Analysis parameters are shown in Table 2.

### 2.4. Numerical Method and Verification

**Figure 4:**
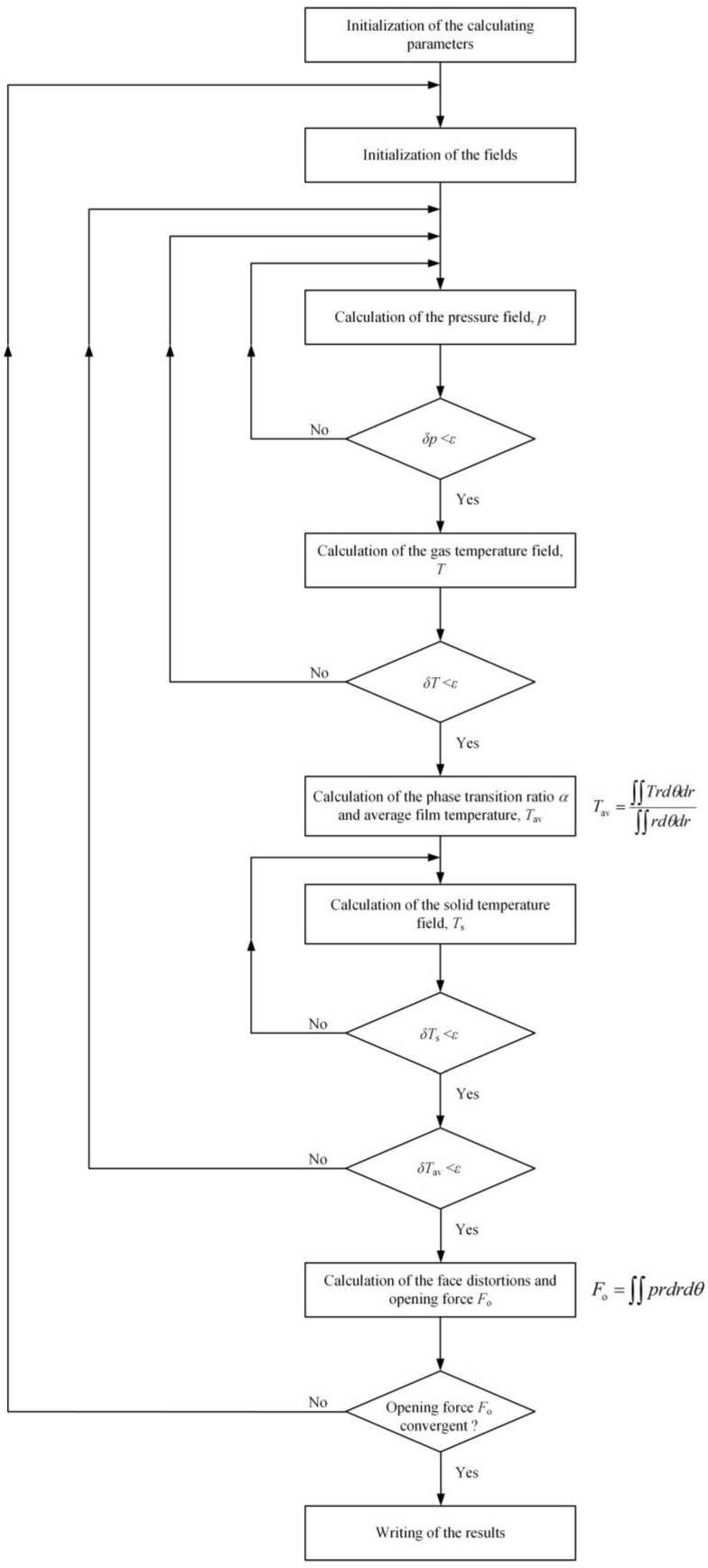
Flowchart of the numerical procedure.

The finite difference method is utilized to obtain the film pressure, film temperature and ring temperature. The finite element method is used for coupling the calculation of the face elastic and thermal distortions [28]. As shown in Figure 4, film pressure, film temperature, vapor condensation, ring temperature, face distortions and seal clearance are successively calculated into four overlapping loops. The entire iterative process is repeated until the convergence criterion on the opening force is satisfied. The value of error limit, *ε*, for convergence criterion is 10^−5^.

**Figure 5:**
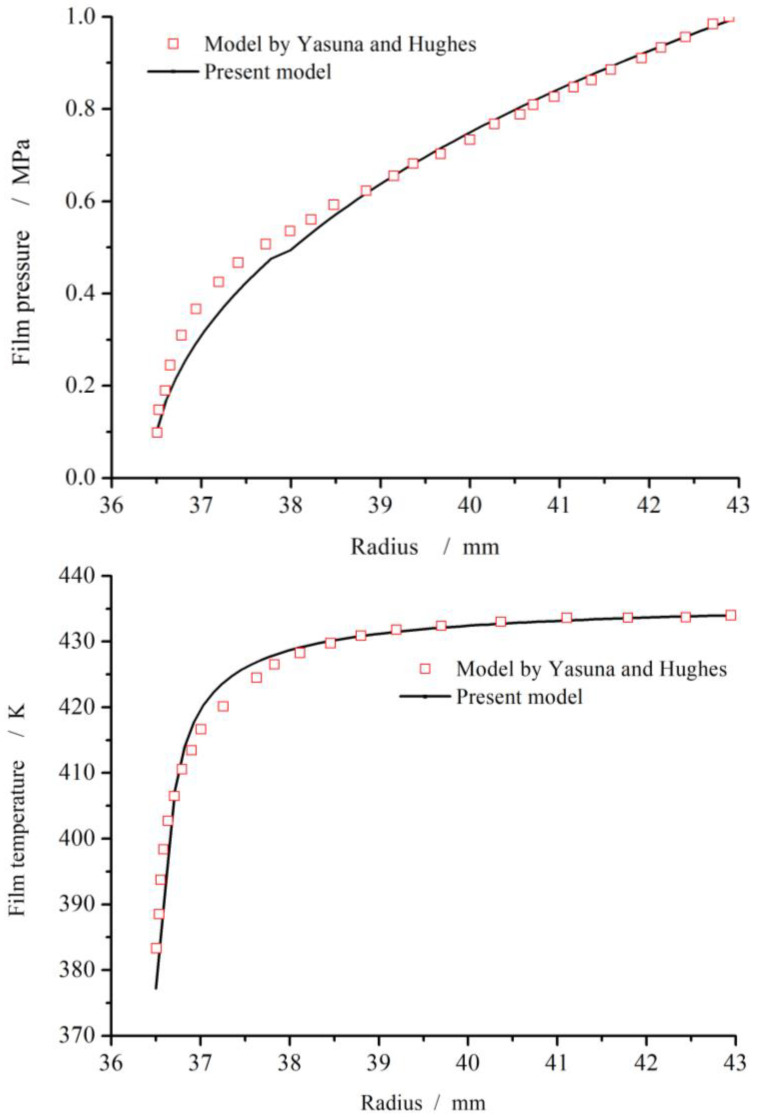
Comparison of film pressure and temperature fields between present model and referenced model [11] (*ω* = 2000 r/min, *h*_0_ = 5 μm, *p*_o_ = 1.0 MPa, *p*_i_ = 0.1 MPa).

In order to validate the model, distributions of film pressure and temperature between the continuous boiling model by Yasuna and Hughes [10] and the present model are compared, for a hot water smooth face seal with inner radius 36.5 mm and outer radius 42.9 mm. For the case of seal pressure 1.0 MPa, clearance 5 μm and inlet vapor temperature 434 K much higher than the saturation temperature, the values of film pressure and temperature obtained by the present model agree well with Yasuna and Hughes’ model as well as the same changing trend, as shown in Figure 5. As a whole, the theoretical results of the present model are in good agreement with Yasuna and Hughes’ work.

## 3. Phase Transform Characteristics on Groove Faces

**Figure 6:**
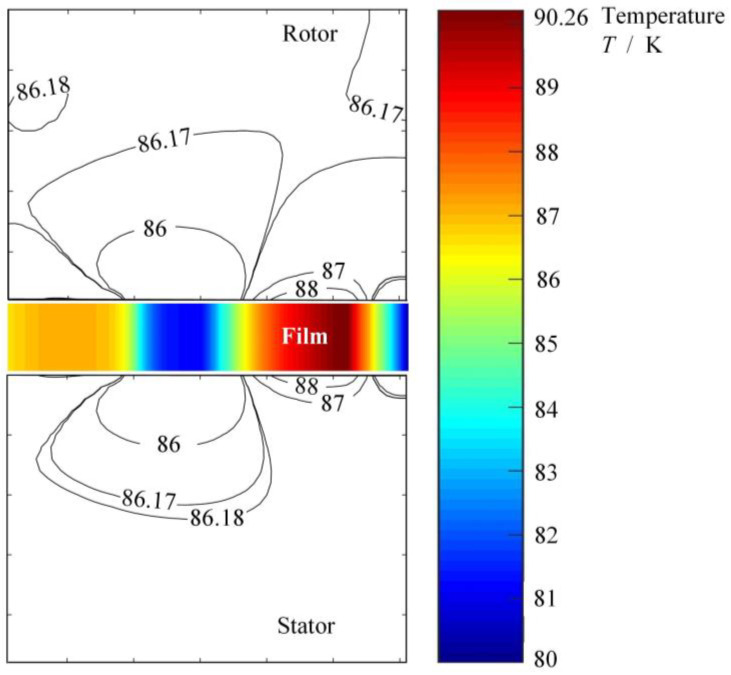
Cross-sectional temperature fields of the seal at *T*_o_ = 80 K, *p*_o_ = 3.1 MPa, *h*_0_ = 2.0 μm, *ω* = 80,000 r/min.

The temperature rise of liquid film caused by viscous shear is the main factor of vaporization. For the non-contact face seal, the temperature rise is also affected by the heat transfer between the film and the seal rings as well as the surface grooves. Figure 6 shows the cross-sectional temperature fields of the seal. As shown in the figure, there is a temperature rise of about 10 K from the inlet to the outlet for the film between the seal faces. Meanwhile, there is a temperature gradient of about 5 K in the stator ring as while as in the rotor ring in the radial direction.

**Figure 7:**
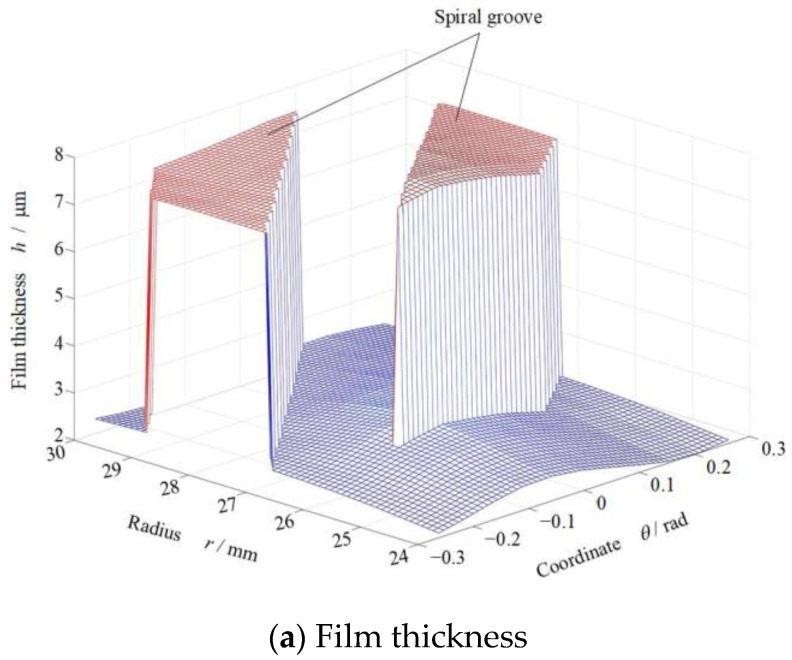

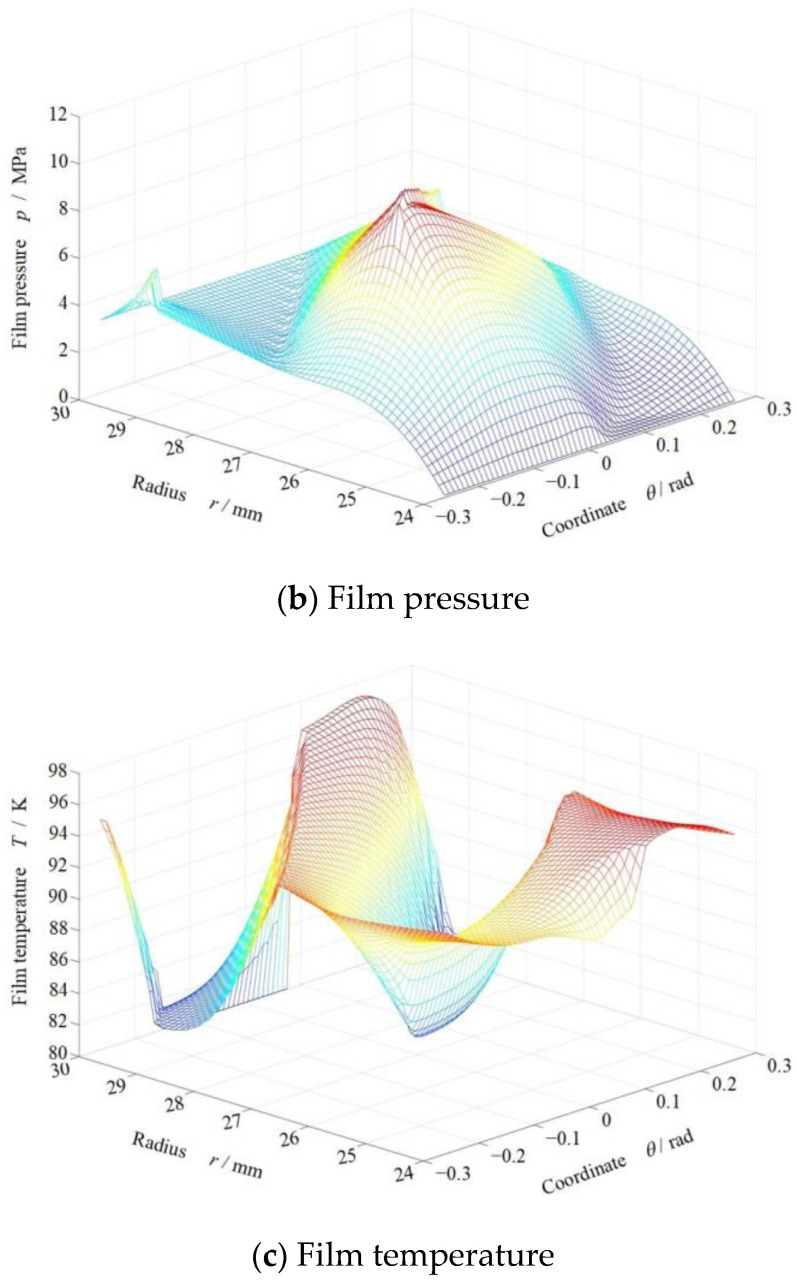
Distributions of film thickness and temperature at *T*_o_ = 80 K, *p*_o_ = 3.1 MPa, *h*_0_ = 2.0 μm, *ω*= 80,000 r/min.

Generally, the temperature gradient in the seal rings produces face distortion, which affects seal performance in return. To analyze the influence of phase transition on sealing performances clearer, the pressure distribution, temperature distribution and film thickness distribution in the sealing clearance is shown in Figure 7.

As shown in Figure 7a, here, a convergent clearance from about 4 μm to 2 μm between the seal faces is occurred due to the pressure and temperature gradient in the seal face along radial direction. In addition, there is a sinusoidal half-wave deformation along the circumference at the inner diameter with an amplitude of about 0.5 μm.

**Figure 8:**
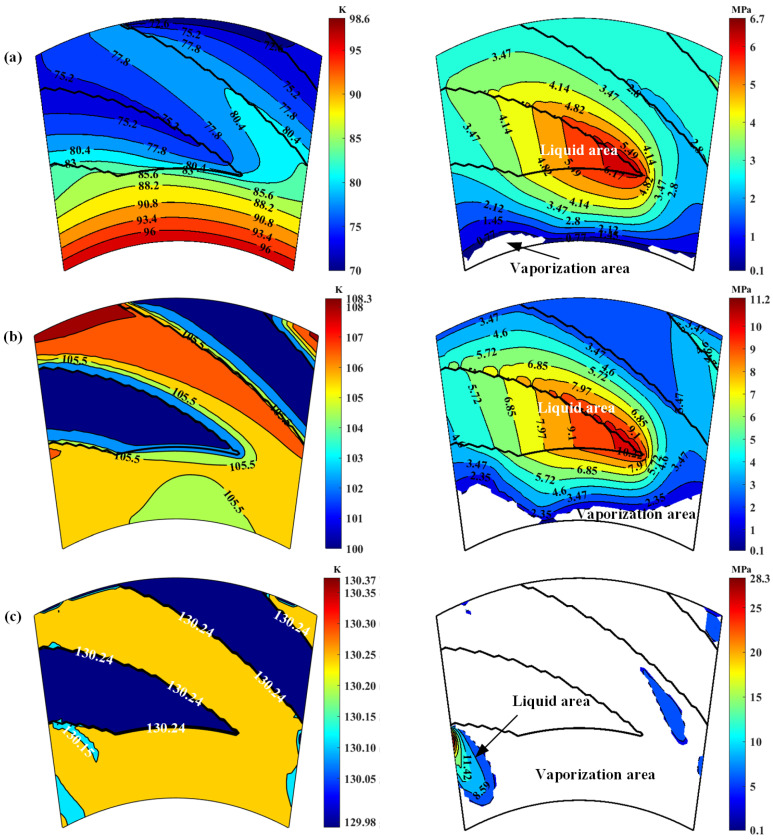
Film temperature and phase transform distributions at *p*_o_ = 3.1 MPa, *h*_0_ = 2.0 μm, *ω* = 80,000 r/min: (**a**) *T*_o_ = 70 K; (**b**) *T*_o_ = 100 K; (**c**) *T*_o_ = 130 K.

Figure 7b gives the pressure distribution of fluid film, the maximum pressure is about 7 MPa occurred in the root of spiral groove, and the pressure decreases to 0.1 MPa at the inner diameter. According to Figure 7b,c, we can also see in the figure that the vaporization exists in the seal face at the inside diameter,

The more important is that, with increase in temperature and decrease in pressure, the liquid film tends to vaporization according to Figure 7, especially at the low-pressure region of the seal face. Further, Figure 8 shows the film temperature and phase transform distributions at the seal faces with increase in seal temperature. Clearly, the phase transform happens mainly in the low-pressure region of the sealing faces. This because that the film pressure keeps dropping when liquid lowing from high pressure side to the low-pressure side. Once the film pressure reaches lower than the saturated vapor pressure, the phase transition from liquid to gas may be happens. However, the seal temperature has a significant influence on the phase transition, with increase in seal temperature from 70 K to 130 K, the vaporization area of the sealing zone develops rapidly from almost zero to almost complete.

It should also be noted that, the spiral grooves lead to a very complex distribution of vaporization. The region of vaporization cannot be simply divided by vaporization radius. As shown in Figure 8, vaporization often occurs in parts of the low-pressure side first, and its distribution is irregular. With the increase in temperature, phase transition occurs throughout the sealing surface, especially in the slotted area.

**Figure 9:**
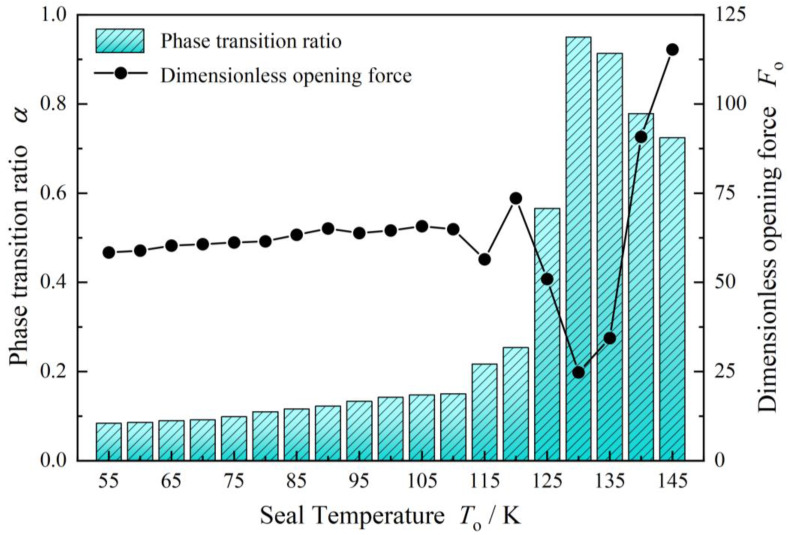
Variation of opening force and phase transition ratio with increasing temperature (*p*_o_ = 3.1 MPa, *h*_0_ = 2.0 μm, *ω* = 80,000 r/min).

Theoretically, due to irregular pressure distribution and temperature distribution, the vaporization phase transition may result in unstable sealing performance. As shown in Figure 9, the opening force increases firstly with increasing seal temperature after 110 K and reaches a peak value at about 120 K. Further, when the seal temperature exceeds 130 K, the opening force increases greatly. As a whole, in the seal temperature range from 55 K to 140 K, the opening force varies by 200%. At the same time, the leakage rate continues to decrease until the seal temperature reaches to about 120 K. Then, the leakage rate increases quickly with increasing seal temperature.

**Figure 10:**
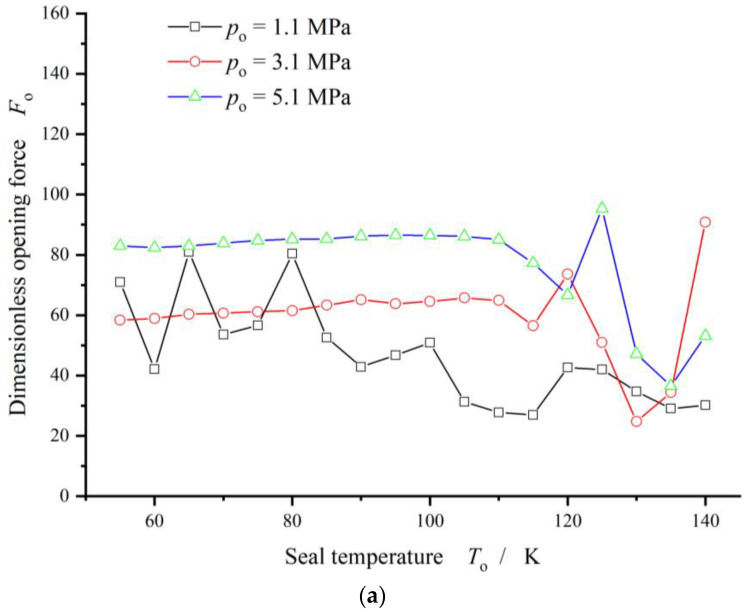

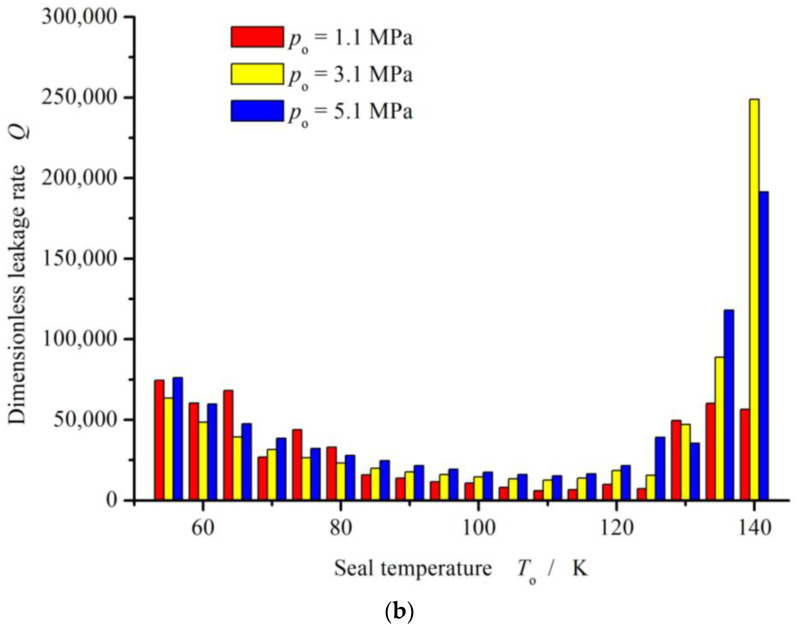
Variation of sealing performance with increasing seal temperature with consideration of phase transform (*p*_o_ = 3.1 MPa, *h*_0_ = 2.0 μm, *ω* = 80,000 r/min). (**a**) Opening force, (**b**) Leakage rate.

Another important conclusion is that, as shown in Figure 10, when the seal temperature exceeds 110 K, the phase transition ratio increases and varies quickly with increasing seal temperature for the liquid oxygen face seal, which leads to dramatic fluctuation of the opening force, meaning the seal is in an unstable state, which may cause the seal to “suddenly burst” and excessive leakage, or lead to catastrophic collapse, making the seal in an unstable state as discussed in contact face seals [9].

## 4. Sealing Performance

In this section, the influence of the vaporization phase transition on the sealing performance is discussed under cryogenic conditions with different speed, seal pressure and clearance.

### 4.1. Seal Pressure

**Figure 11:**
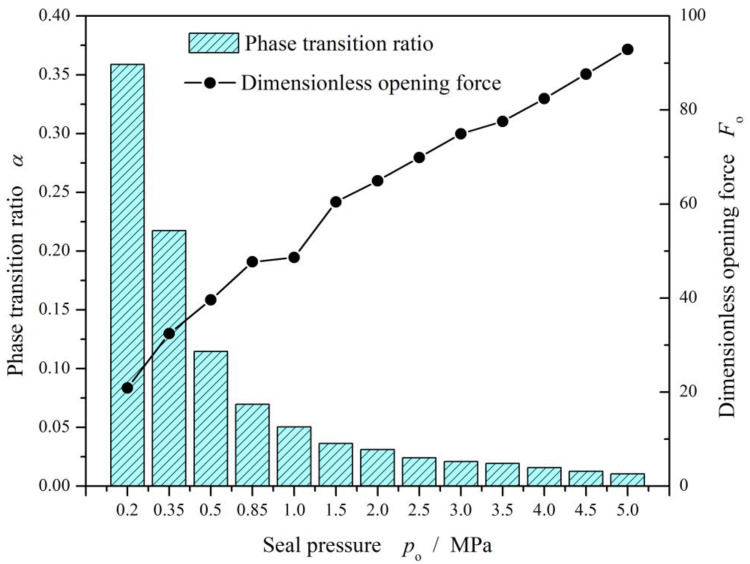
Change in opening force and phase transition ratio with increasing seal pressure (*T*_o_ = 70 K, *h*_0_ = 2.0 μm, *ω* = 50,000 r/min).

Figure 11 gives the curves of opening force and vaporization rate. Obviously, the vaporization rate decreases monotonically and quickly from 35% to 3% with the seal pressure increases from 0.2 MPa to 1.5 MPa in the case of seal temperature 70 K. Meanwhile, the opening force increases about four times.

**Figure 12:**
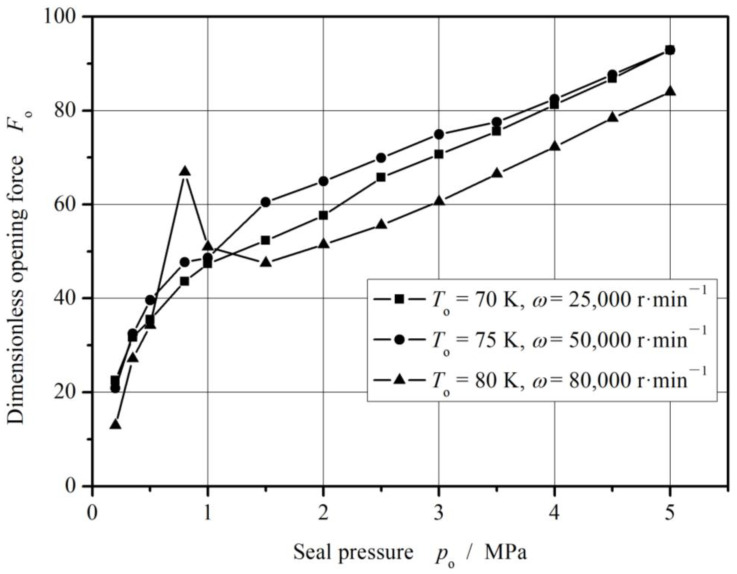

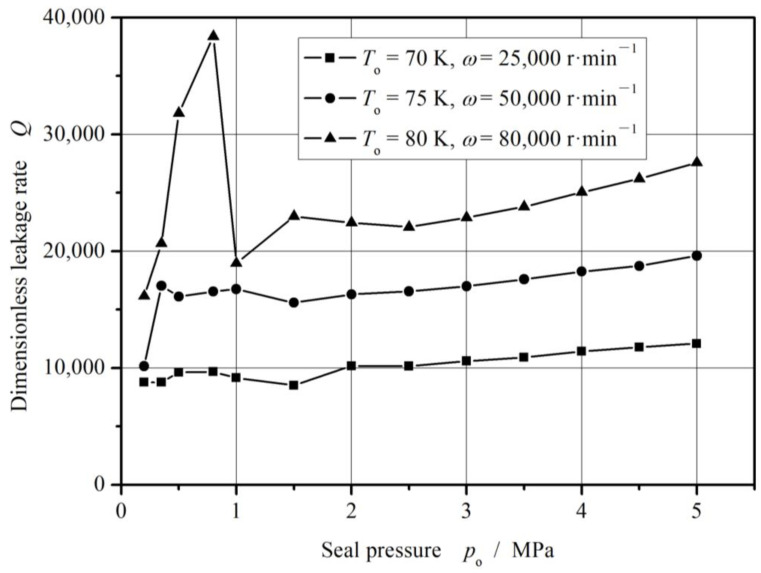
Sealing performance with increasing seal pressure.

Figure 12 illustrates the opening force and leakage rate with increasing seal pressure with consideration of the vaporization phase transition. In a whole, the seal pressure present monotonical and significant influence on the opening force and leakage rate. The opening force as well as the leakage rate increases with increasing seal pressure.

However, when the seal pressure is lower than 1.0 MPa at the seal temperature 70 K, the opening force and the leakage rate fluctuate obviously as shown in Figure 12, the more than 50%. For seal design, this pressure and temperature zone is an unstable zone that should be avoided.

### 4.2. Rotational Speed

**Figure 13:**
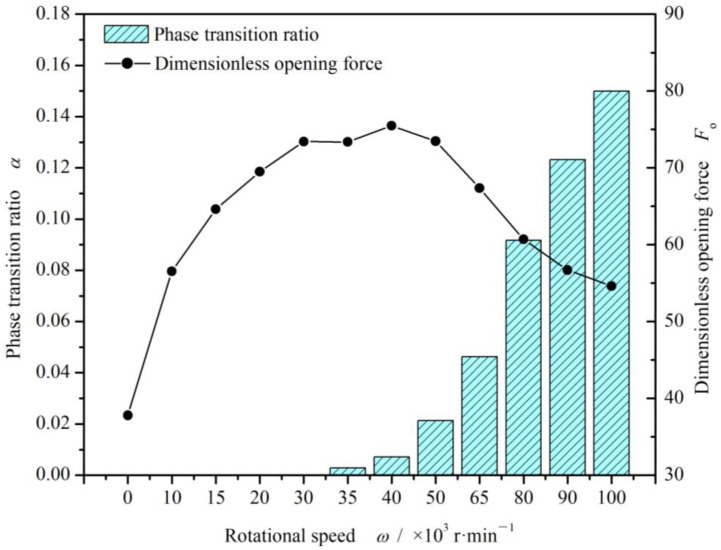
Change in opening force and cavitation ratio with increasing rotational speed (*T*_o_ = 70 K, *p*_o_ = 3.1 MPa and *h*_0_ = 2.0 μm).

As shown in Figure 13, once the vaporization phase transition happens, the opening force begins to decrease. The reason may be that, the vaporization results in a decrease in the viscosity of the fluid, which leads to a decrease in the hydrodynamic effect.

**Figure 14:**
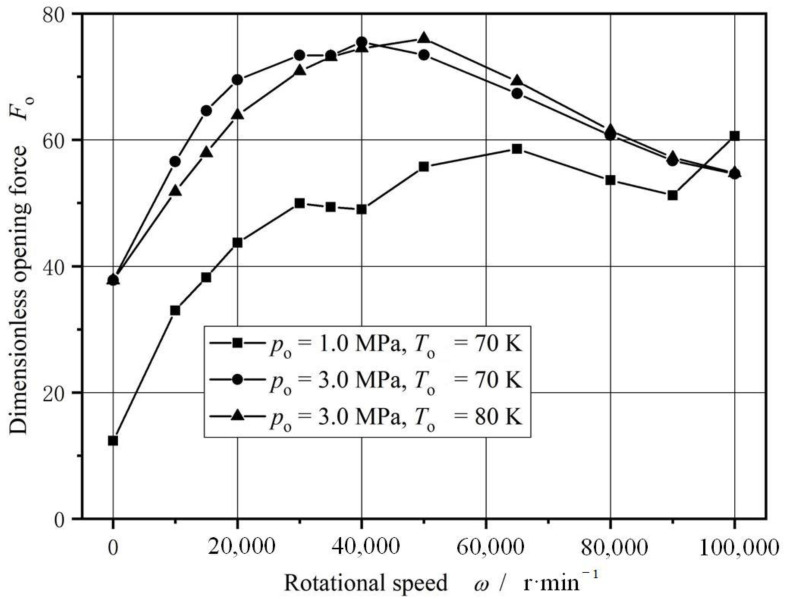

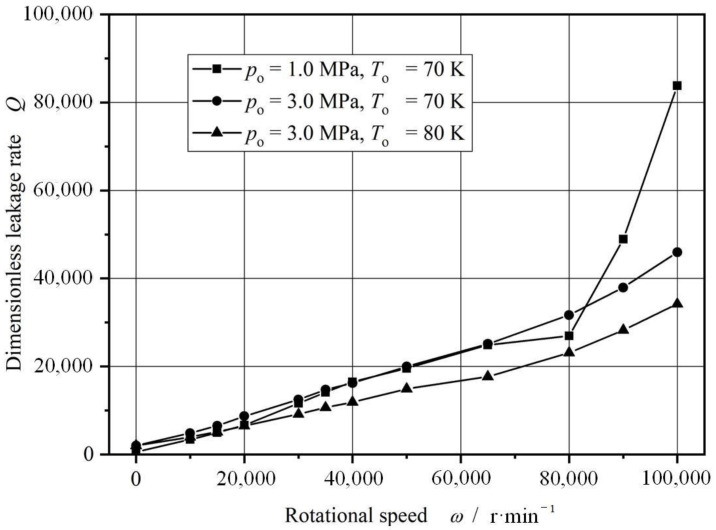
Sealing performance with increasing rotational speed.

Figure 14 gives the curves of opening force and leakage rate under different speed. Obviously, the opening force reaches maximum values in a rotational speed range from 3000 r/min to 4000 r/min. The increase ratio of the opening force may reach 400%. However, the opening force keeps dropping with increasing speed after 4000 r/min.

In addition, the leakage rate keeps increasing with increase in rotational speed, even after the vaporization phase transition happens. This is because that, the vaporization causes the viscosity of the fluid to decrease, which in turn reduces the leakage flow resistance.

### 4.3. Seal Clearance

**Figure 15:**
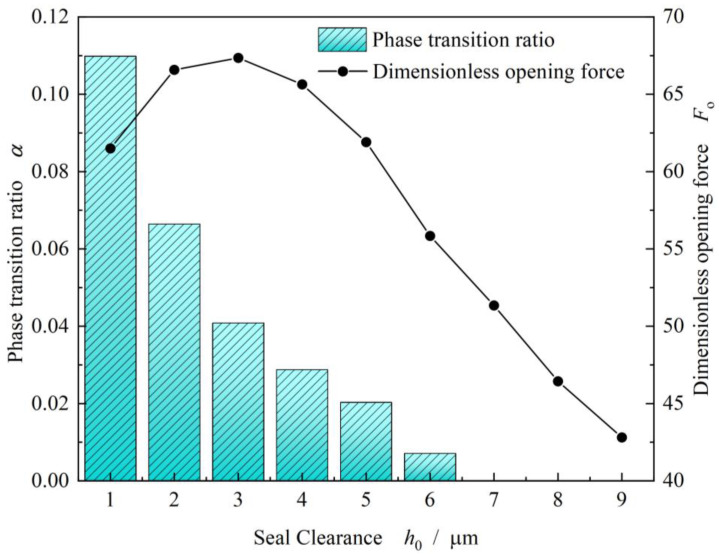
Change in opening force and phase transition ratio with increasing clearance (*T*_o_ = 80 K, *p*_o_ = 3.1 MPa, *ω* = 80,000 r/min).

As discussed in above, the weakening of vaporization leads to increase in opening force, while the increase in clearance often results in increase in opening force due to lower shear effect. So, the opening force presents a peak value at about 2.5 μm as shown in Figure 15. But, as a whole, the opening force and the vaporization ratio both decrease with increasing clearance.

**Figure 16:**
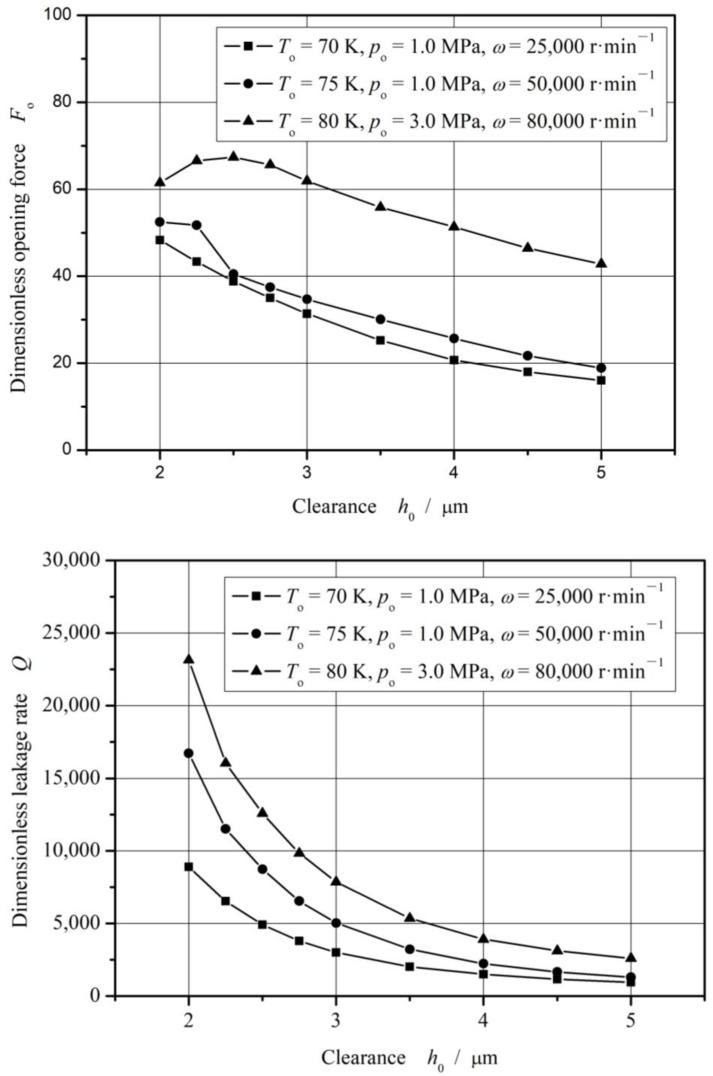
Sealing performance with increasing clearance.

Figure 16 illustrates the opening force and leakage rate with increasing clearance with consideration of the vaporization phase transition. As can be seen, when the clearance is greater than 3 mm, both the opening force and the leakage rate present monotone decreasing trend.

**Figure 17:**
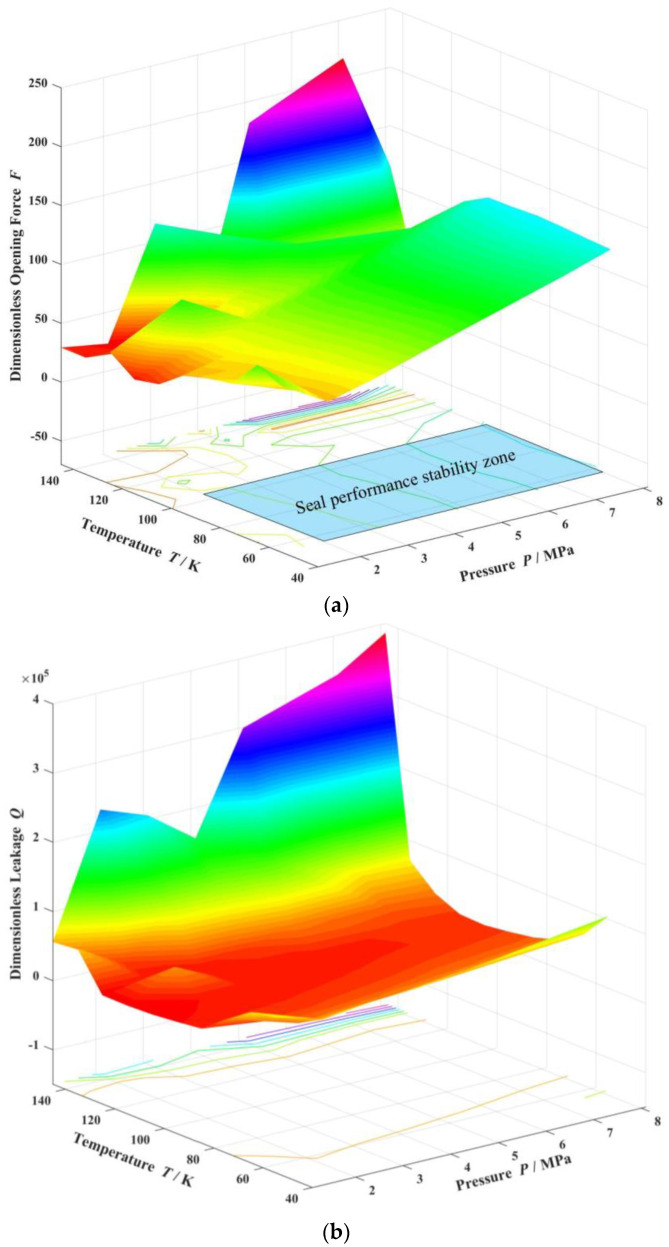
Variation of opening force and leakage rate in cryogenic liquid oxygen region (*h*_0_ = 2.0 μm, *ω* = 80,000 r/min). (**a**) Opening force, (**b**) Leakage rate.

Furthermore, the variation of opening force and leakage rate with increases in both temperature and pressure in the cryogenic liquid oxygen region is shown in Figure 17. Clearly, the opening force remains relatively stable with increasing temperature far from the saturation temperature in the cryogenic liquid region but varies and increases sharply close to the saturation temperature as discussed previously. Correspondingly, the leakage rate also varies sharply and increases rapidly near the saturation temperature point. This means that it is difficult to keep the seal operating steadily near saturation point. According to the figure, it may be concluded that, the seal runs stable in a region with temperature ranging from 55 K to 100 K and pressure ranging from 2 MPa to 8 MPa.

## 5. Conclusions

(a) A numerical model based on the saturated vapor pressure is established to investigate the vaporization phase transition property of liquid oxygen sealing film, with consideration of heat transfer as well as face distortions. Distributions of vaporization phase transition for cryogenic liquid oxygen are obtained in spiral groove face seals.
(b) Spiral grooves on gas face seals make film temperature distribution and vaporization distribution more uniform at groove region. Meanwhile, with increase in seal temperature and decrease in seal pressure, the vaporization area extends from the low-pressure side to the grooves are, and the vaporization rate increases rapidly.
(c) For cryogenic liquid oxygen spiral groove face seals, vaporization brings drastic fluctuation and non-monotonic change in opening force. With the increase in seal temperature from 55 K to 140 K, the opening force fluctuates violently, and the fluctuation range is more than 50%, showing obvious instability. There is a range of pressure and temperature values, the seal can be stable operation.

## Figures and Tables

**Table 1:** Structural features of the spiral groove gas face seal.

Item	Symbol	Dimensions and Data
Inside radius	*r*_i_	24 mm
Outside radius	*r*_o_	30 mm
Spiral radius	*r*_p_	26.5 mm
Ring thickness	*h*_1_, *h*_2_	15 mm
Groove depth	*h*_d_	5 μm
Groove number	*N*	12
Spiral angle	*β*	16°

**Table 2:** Characteristics of the ring materials.

Characteristics	Carbon	Steel
Density (kg m^−3^)	1800	7930
Young’s modulus (GPa)	25	204
Poisson’s coefficient	0.2	0.3
Specific heat capacity (J Kg^−1^K^−1^)	710	500
Thermal conductivity (W m^−1^ K^−1^)	129	17
Linear thermal expansion coefficient (10^−6^ °C)	4.0	16.0

## Data Availability

Data are contained within the article.
